# Effect of exogenous microorganisms on the fermentation quality, nitrate degradation and bacterial community of sorghum-sudangrass silage

**DOI:** 10.3389/fmicb.2022.1052837

**Published:** 2022-11-01

**Authors:** Meirong Zhao, Hongyu Zhang, Gang Pan, Hang Yin, Juanjuan Sun, Zhu Yu, Chunsheng Bai, Yanlin Xue

**Affiliations:** ^1^College of Horticulture, Shenyang Agricultural University, Shenyang, China; ^2^Institute of Grassland Research, Chinese Academy of Agricultural Sciences, Hohhot, China; ^3^College of Grassland Science and Technology, China Agricultural University, Beijing, China; ^4^Inner Mongolia Engineering Research Center of Development and Utilization of Microbial Resources in Silage, Inner Mongolia Academy of Agriculture and Animal Husbandry Science, Hohhot, China; ^5^Inner Mongolia Key Laboratory of Microbial Ecology of Silage, Inner Mongolia Academy of Agriculture and Animal Husbandry Science, Hohhot, China

**Keywords:** sorghum-sudangrass silage, fermentation quality, nitrate, *Lactobacillus buchneri*, *Lactobacillus brevis*, *Bacillus subtilis*

## Abstract

This study aims to investigate the effects of adding *Lactobacillus buchneri* (LB), *Lactobacillus brevis* (LBR) and *Bacillus subtilis* (BS) on the fermentation quality, nitrate degradation and bacterial community of sorghum-sudangrass silage. The results showed that the addition of LB significantly increased the pH and acetic acid content (*p* < 0.05), but high-quality silage was obtained. The addition of LBR and BS improved the fermentation quality of sorghum-sudangrass silage. The use of additives reduced the nitrate content in sorghum-sudangrass silage. The LB group increased the release of N_2_O at 3–7 days of ensiling (*p* < 0.05), and LBR and BS increased the release of N_2_O at 1–40 days of ensiling (*p* < 0.05). On the first day of ensiling, all silages were dominated by *Weisslla*, over 3 days of ensiling all silages were dominated by *Lactobacillus*. *Acinetobacter*, *Serratia*, *Aquabacterium,* and *unclassified_f_enterobacteriaceae* showed significant negative correlations with nitrate degradation during sorghum-sudangrass ensiling (*p* < 0.05). The BS and LBR groups increased the metabolic abundance of denitrification, dissimilatory nitrate reduction, and assimilatory nitrate reduction (*p* < 0.05). Overall, the additive ensures the fermentation quality of sorghum-sudangrass silage and promotes the degradation of nitrate by altering the bacterial community.

## Introduction

With the development of the livestock industry and increasing consumer demand for meat and dairy products, there is a significant demand for the production of silage, and statistics show that China produces greater than 280 million tons of silage annually, especially in some arid areas where there is an increasing demand for high-yielding and water-saving fodder crops (Fijalkowska et al., 2020). Due to its excellent characteristics of high drought resistance, high water use efficiency, and high biological yield, sorghum-sudangrass (*Sorghum bicolor×Sorghum sudanense*) has become one of the high-quality modulated silage raw materials in arid areas (McCary et al., 2020). However, sorghum-sudangrass easily accumulates nitrate forage (Holman et al., 2019). Stress conditions such as drought and low temperature during its growth, and excessive use of nitrogen fertilizer in pursuit of high yield can affect the level of nitrate accumulation in sorghum-sudangrass (Crawford et al., 1961; Harada et al., 2000; Holman et al., 2019). Nitrate in silage can threaten animal health, the safety of milk, and other animal foods safety and even cause significant economic losses in severe cases (Vermunt and Visser, 2011; Schrenk et al., 2020). The high nitrate content in sorghum-sudangrass silage limits its use as animal feed. An urgent issue that needs to be resolved: is the reduction of nitrate content in sorghum-sudangrass silage.

Ensiling can reduce nitrate in sorghum-sudangrass to some extent content (Spoelstra, 1987; Bai C. et al., 2022). During the ensiling process, nitrate is degraded to produce the intermediate product nitrite, and the end products nitric oxide, ammonia, and nitrous oxide (Spoelstra, 1985). However, the nitrate content in silage may still threaten the health of ruminants by exceeding the safe limit of nitrate in forage (Spoelstra, 1983; Bai C. et al., 2022). In response, researchers have tried to use silage additives to regulate the nitrate content in silage. Takayoshi et al. (1980) used glucose as a silage additive to regulate the nitrate content in Italian ryegrass silage. Li et al. (1992) investigated the effect of ammonium hydroxide and calcium carbonate on the degradation of nitrate in corn silage. In the above studies, nitrates were degraded during ensiling after using chemical additives for silage preparation, but the quality of the silage was reduced. Today, producers often use microbial inoculants to regulate the silage fermentation process (Muck et al., 2018; Romero et al., 2018). Tian et al. (2021) demonstrated that *Bacillus subtilis* promoted fermentation quality and nutrient composition by altering bacterial communities and metabolic profiles. Xie et al. (2021) showed that the use of lactic acid bacteria to prepare sorghum silage improved fermentation quality. The ability of lactic acid bacteria and *Bacillus subtilis* to degrade nitrate has been demonstrated in studies on kimchi, whereas the use of those bacteria has rarely reported in research on silage fermentation (Sun et al., 2016; Zhang et al., 2022). Based on this, we hypothesized that inoculation lactic acid bacteria and *Bacillus subtilis* prior to sorghum-sudangrass silage would both enhance fermentation quality and promote nitrate degradation. However, experimental evidence is lacking to support this hypothesis.

Ensiling is a complex microbial fermentation process in which microorganisms interact with each other and with their environment during fermentation (Xu et al., 2019; Ding et al., 2020). Previous experimental cultivation studies have shown that microorganisms, such as *Enterobacteriaceae* and *Lactobacillus*, are involved in nitrate degradation (Spoelstra, 1985). Cultivation experiments are commonly applied in ensiling experiments, but microbial communities often contain a large number of bacteria that cannot be cultivated under laboratory conditions (Eikmeyer et al., 2013). Therefore, the complex microbial communities, microbial interactions, and their functions during nitrate degradation in silages have not been thoroughly studied in the previous experiments. In recent years, changes in silage fermentation parameters and their bacterial community dynamics during silage have been gradually analysed, with the application of molecular techniques in the field of silage (Xu Z. et al., 2017; You et al., 2021; Bai J. et al., 2022). This methodology provides the possibility to deeply explain the nitrate degradation during silage. For example, Bai C. et al. (2022) used high-throughput sequencing to illustrate the effects of *Panobacter* spp., *Pseudomonas spp*., and *Enterobacter* spp. on the nitrate content of silage sorghum-sudangrass. However, there are few studies on the effects of the use of *Lactobacillus* and *Bacillus subtilis* as additives for sorghum-sudangrass on nitrate content, nitrate degradation, and the correlation between nitrate content and silage fermentation quality and microbial community changes. Therefore, in this study, *Lactobacillus buchneri*, *Lactobacillus brevis*, and *Bacillus subtilis* were used as additives with the aim of investigating their role in silage with respect to fermentation quality and nitrate degradation in sorghum-sudangrass.

## Materials and methods

### Silage preparation

Sorghum-sudangrass (Jicao No.6) was grown at the experimental farm of Shenyang Agricultural University (123^°^ 25′ E, 41^°^ 46′ N) and harvested at the heading stage, leaving a stubble height of 15–20 cm. After harvest, the sorghum-sudangrass was chopped into 1–2 cm lengths using a grass shredding machine (Donghong No. 1, Donghong Mechanical Equipment Co., Ltd., China). Silage were treated with the following: sterile water control (CK), 2.0 × 10^5^ colony-forming units (CFU)/g of *Lactobacillus buchneri* (LB, Gansu Pro-Bicon Biotech Co., Ltd., China), 1.0 × 10^5^ colony-forming units (CFU)/g of *Lactobacillus brevis* (LBR, Laboratory of China Agricultural University) and 2.0 × 10^6^ colony-forming units (CFU)/g of *Bacillus subtilis* (BS, Laboratory of Shenyang Agricultural University). The additives were dissolved in 5 ml of sterile water, and then each 5 ml of bacterial solution was sprayed onto 1 kg of fresh matter (FM). An equal amount of sterile water was added to the control group. After thoroughly mixing, 230 g of treated material was placed in anaerobic jars (300 ml; Saipu Instrument Technology Co., Ltd., Zhenjiang, China). A total of 60 jars (four treatments × five ensiling durations × three replicates) were prepared and stored at room temperature (25–30°C). Each treated silage was sampled after storage for 1，3, 7, 15, and 40 days for determining the fermentation quality, nutrient composition, microbial counts, and microbial community. In addition, the fermentation gas was collected at each sampling time point in a 500 ml aluminum foil gas sampling bag (Dalian Delin Co., Ltd., China) for N_2_O determination.

### Analyses of silage fermentation quality and nutrient composition

To determine the fermentation traits of forage, a sample (10 g) of silage was mixed with 90 g of deionized water and kept in a refrigerator at 4°C for 24 h. The liquid extract was filtered through four layers of cheesecloth and filtered paper. Then, the filtrates were used to measure pH, ammonia nitrogen (NH_3_-N), and organic acids. The pH value of the extracts was determined using a pH meter (PB-10, Sartorius Group, Göttingen, Germany). Organic acids and nitrates were determined by high-performance liquid chromatography (Bai C. et al., 2022). The concentrations of N_2_O in the gas samples were analysed by gas chromatograph (Agilent 7890A, Agilent Technologies Limited Co., United States) within a week (Xu T. W. et al., 2017). A representative silage sample of 10 g was obtained, added to 90 ml of distilled water, shaken at 180 rpm for 30 min, and then the solution was diluted in a gradient. Microbial counts were analysed using the plate count method on Man Rogosa Sharpe agar, Violet Red Bile agar, and Rose Bengal agar (Beijing Aoboxing Bio-tech Co. Ltd., Beijing, China) as reported by Cai et al. (1999).

The dry matter (DM) of the fresh sorghum-sudangrass and silage was determined by oven drying at 65°C for 48 h. Colorimetry after reaction with anthrone reagent was used to determine the water-soluble carbohydrate (WSC) content (Murphy, 1958). The crude protein (CP = total N × 6.25) was determined using a Kjeldahl apparatus (Kjeltec 8,400; FOSS Co. Ltd., Hillerød, Denmark), according to previously published work (AOAC, 1990). The neutral detergent fiber (using heat-stable α-amylase, NDF) and acid detergent fiber (ADF) were measured using an Ankom 2000 fiber analyser (Ankom, Macedon, NY, United States), according to Van Soest’s procedures (Van Soest et al., 1991).

### Bacterial community analysis

A 10 g fresh sample was placed into 90 ml of sterile distilled water. The mixture was placed in a low-temperature oscillator (THZ-98C, Shanghai Yiheng Scientific Instrument Co., Ltd., Shanghai, China) at 4°C, 180 rpm/min for 30 min and then subject to filtration through 4 layers of sterile gauze. The filtrate was centrifuged in a cryogenic centrifuge (ST 16R, Thermo Fisher Scientific, Inc., Waltham, United States) at 8000 rpm/min at 4°C for 15 min to enrich the sediment. Sediments were used for high-throughput sequencing (Wang et al., 2018).

Bacterial community genomic DNA was extracted from silage samples using the FastDNA® SPIN Kit and the FastPrep® Instrument (MP Biomedicals, Santa Ana, CA) according to the manufacturer’s instructions. The hypervariable region V3-V4 of the bacterial 16S rRNA gene was amplified with the primer pairs 341F (5′- CCTAYGGGRBGCASCAG-3′) and 806R(5’-GGACTACNNGGGTATCTAAT-3′) using an ABI GeneAmp® 9,700 PCR thermocycler (ABI, CA, United States). The PCR product was extracted from a 2% agarose gel, purified using the AxyPrep DNA Gel Extraction Kit (Axygen Biosciences, Union City, CA, United States) and quantified using a Quantus™ Fluorometer (Promega, United States). Purified amplicons were pooled in equimolar amounts and paired-end sequenced on an Illumina MiSeq PE300 platform (Illumina, San Diego, United States) by Majorbio Bio-Pharm Technology Co. Ltd. (Shanghai, China). The raw 16S rRNA gene sequencing reads were demultiplexed, quality-filtered by fastp version 0.20.0 and merged by FLASH version 1.2.7 based on the following criteria. Operational taxonomic units (OTUs) with a 97% similarity cut-off were clustered using UPARSE version 7.1, and chimeric sequences were identified and removed. The taxonomy of each OTU representative sequence was analysed against the 16S rRNA database using a confidence threshold of 0.7 by RDP Classifier version 2.2. The microbial diversity within an individual sample was assessed using the following alpha diversity indices: Shannon diversity index, Simpson diversity index, Chao richness estimator, Ace richness estimator and Coverage. Beta diversity was analysed to assess the structural variation of the microbiota. Subsequently, principal component analysis (PCoA) was undertaken (Vazquez-Baeza et al., 2013). The heatmap function of R software^2^ and genus information for the silage were used to generate a heatmap. The data were analysed using the free online majorbio cloud platform. The raw sequence data have been uploaded to the sequence read archive (SRA) of the NCBI database (the accession number PRJNA882255).

### Statistical analyses

Factorial analysis of variance was performed to investigate the effects of additives, ensiling duration, and their interactions on the fermentation quality, microbial counts, bacterial community indices, nitrate, and N_2_O content of silage in the General Line Model of SPSS (SPSS 22.0 program, SPSS Inc., Chicago, Illinois, United States). Significant differences were compared using Tukey multiple range tests, and *p* < 0.05 indicated statistical significance.

## Results

### Characteristics of fresh sorghum-sudangrass

The chemical parameters and microbial counts of fresh sorghum-sudangrass are shown in Table 1. The concentrations of WSC, CP, NDF, and ADF in sorghum-sudangrass were 233.00, 86.25, 481.93, and 232.62 g/kg DM, respectively. The nitrate content was 5520.55 mg/kg DM. The counts of undesirable microbes containing coliform bacteria, yeast, and mould were 5.29, 4.93, and 2.58 lg CFU/g FM, respectively, whereas the lactic acid bacteria count was 4.35 lg CFU/g FM.

**Table 1:** Chemical composition and microbial counts in sorghum-sudangrass.

Item	Content
DM (g/kg FM)	217.08 ± 1.77
WSC (g/kg DM)	233.00 ± 12.30
CP (g/kg DM)	86.25 ± 0.60
NDF(g/kg DM)	481.93 ± 2.81
ADF(g/kg DM)	232.62 ± 3.90
Nitrate (mg/kg DM)	5520.55 ± 36.75
Lactic acid bacteria (lg CFU/g FM)	4.35 ± 0.09
Coliform bacteria (lg CFU/g FM)	5.29 ± 0.07
Yeast (lg CFU/g FM)	4.93 ± 0.54
Mould (lg CFU/g FM)	2.58 ± 0.49

DM, dry matter; FM, fresh matter; WSC, Water soluble carbohydrate; CP, crude protein; NDF, neutral detergent fiber; ADF, acid detergent fiber; CFU, colony-forming units.

### Fermentation quality and nutrient composition of silage

Additives significantly affected pH, lactic acid (LA), and acetic acid (AA) concentrations and lactic acid bacteria counts in sorghum-sudangrass silage (*p* < 0.05; Table 2). The pH of the LB group was significantly lower than that of CK at 3 days of ensiling; however, it was significantly higher than that of CK at 40 days of ensiling (*p* < 0.05). The AA content of the LB group increased rapidly during 15–40 days of ensiling and was significantly higher than that of CK at 40 days of ensiling (*p* < 0.05). The LBR group had a significantly lower pH than CK after 3 days of ensiling (*p* < 0.05). Compared to CK, the BS group had lower pH and higher LA content on days 3 and days 40 of ensiling (*p* < 0.05). The AA content of the LBR and BS group was significantly lower than that of CK at 40 days of ensiling (*p* < 0.05). Butyric acid was not detected during the fermentation process. After 40 days of ensiling, the NH_3_-N content of all silages was in the range of 4.88–5.31. The number of lactic acid bacteria reached the highest in all silages at 7 days (*p* < 0.05). At 40 days, the number of lactic acid bacteria in the LBR and BS groups was significantly lower than that of CK, and the number of lactic acid bacteria in the LB group was significantly higher than that in CK (*p* < 0.05). On the first day of ensiling, the numbers of coliform bacteria and yeast were significantly higher in the LBR and BS groups compared with CK (*p* < 0.05), and the number of coliform bacteria and yeast in the LB group was similar to that in CK (*p >* 0.05). However, as the number of ensiling days increased, coliform bacteria and yeast, which are harmful to silage, were not detected in all silages.

**Table 2:** Fermentation quality and microbial of sorghum-sudangrass silage.

Item	Ensiling days	Treatment				SEM	Significance		
		CK	LB	LBR	BS		*T*	*D*	*T* × *D*
pH	1	4.14a	4.14a	4.19a	4.19a	0.003	<0.001	<0.001	<0.001
	3	3.97Ab	3.90Bb	3.76Cb	3.78Cb				
	7	3.62Ac	3.64Ac	3.59Bc	3.63Ac				
	15	3.54ABd	3.56Ad	3.51Cd	3.52BCd				
	40	3.51 Bd	3.53Ad	3.45Ce	3.47Ce				
LA (g/kg DM)	1	36.63c	39.26d	38.29c	35.19c	0.499	<0.001	<0.001	0.030
	3	46.59Bb	52.13Bc	64.22Ab	61.16Ab				
	7	82.47a	80.43b	86.68a	83.98a				
	15	78.67Ba	82.53ABab	87.28Aa	84.22ABa				
	40	82.73Ba	84.83Ba	92.61Aa	89.79Aa				
AA (g/kg DM)	1	9.81b	10.41d	10.17b	8.68d	0.024	<0.001	<0.001	<0.001
	3	12.09ABab	13.10Acd	10.83Bb	10.37Bcd				
	7	13.03ab	14.01bc	12.30ab	12.68ab				
	15	14.68ab	16.34b	11.34b	11.57bc				
	40	17.83Ba	27.23Aa	13.73Ca	14.51Ca				
BA (g/kg DM)	1	ND	ND	ND	ND	—	—	—	—
	3	ND	ND	ND	ND				
	7	ND	ND	ND	ND				
	15	ND	ND	ND	ND				
	40	ND	ND	ND	ND				
NH_3_-N %TN	1	0.99c	1.78b	1.69c	1.45d	0.087	0.915	<0.001	0.372
	3	1.98bc	1.83b	2.07c	2.04 cd				
	7	2.68b	2.22b	3.06b	2.74bc				
	15	4.61a	3.90a	3.23b	3.32b				
	40	5.00a	4.88a	5.31a	5.23a				
Lactic acid bacteria	1	8.49Ab	8.49Ab	8.32Bc	8.37Bc	0.029	<0.001	<0.001	<0.001
(lg CFU/g FM)	3	8.28Bc	8.52ABb	8.71Ab	8.67Ab				
	7	9.03a	8.99a	9.08a	9.14a				
	15	8.49Ab	8.64Ab	8.04 Bd	8.05 Bd				
	40	7.86 Bd	7.95Ac	6.35De	6.80Ce				
Coliform bacteria	1	4.70C	5.18 BC	5.32AB	5.84A	—	—	—	—
(lg CFU/g FM)	3	3.26	<2.00	<2.00	<2.00				
	7	<2.00	<2.00	<2.00	<2.00				
	15	<2.00	<2.00	<2.00	<2.00				
	40	<2.00	<2.00	<2.00	<2.00				
Yeast	1	5.57B	5.50B	5.98A	5.96A	—	—	—	—
(lg CFU/g FM)	3	<2.00	<2.00	<2.00	<2.00				
	7	<2.00	<2.00	<2.00	<2.00				
	15	<2.00	<2.00	<2.00	<2.00				
	40	<2.00	<2.00	<2.00	<2.00				
Mould	1	<2.00	<2.00	<2.00	<2.00	—	—	—	—
(lg CFU/g FM)	3	<2.00	<2.00	<2.00	<2.00				
	7	<2.00	<2.00	<2.00	<2.00				
	15	<2.00	<2.00	<2.00	<2.00				
	40	<2.00	<2.00	<2.00	<2.00				

LA, Lactic acid; AA, Acetic acid; BA, Butyric acid; NH_3_-N, ammonia nitrogen; DM, dry matter; FM, fresh matter; TN, total N; CFU, colony-forming units; CK, control; LB, Lactobacillus buchneri; LBR, Lactobacillus brevis; BS, Bacillus subtilis. Different capital letters indicate differences between treatment groups at the same time, different lowercase letters indicate different silage times for the same treatment at the 0.05 level; no letter mean no significant difference. SEM, Standard error of the mean; T, treatment; D, Ensiling days; T × D, The interaction between treatment and ensiling days; ND, Not detected.

The nutrient composition of sorghum-sudangrass at 40 days of silage is listed in Table 3. The CP content of the BS group was significantly higher than that of CK after 40 days of ensiling (*p* < 0.05). Different additives did not affect the DM, WSC, NDF, and ADF contents of sorghum-sudangrass silage.

**Table 3:** Nutrient composition of sorghum-sudangrass silage.

Item	Treatment				SEM	*P*-value
	CK	LB	LBR	BS		
DM (g/kg FM)	214.34	215.53	219.54	217.27	1.290	0.606
WSC (g/kg DM)	25.66	20.95	25.69	21.64	1.501	0.605
CP (g/kg DM)	90.22 BC	93.90AB	89.67C	97.24A	1.079	0.004
NDF(g/kg DM)	463.32	467.86	486.28	476.20	3.594	0.089
ADF (g/kg DM)	226.68	214.25	225.30	219.18	3.165	0.541

DM, dry matter; FM, fresh matter; WSC, Water soluble carbohydrate; CP, crude protein; NDF, neutral detergent fiber; ADF, acid detergent fiber; CK, control; LB, Lactobacillus buchneri; LBR, Lactobacillus brevis; BS, Bacillus subtilis. Different capital letters indicate differences between treatment groups; no letter mean no significant difference. SEM, Standard error of the mean.

### Bacterial community in silage

**Figure 1:**
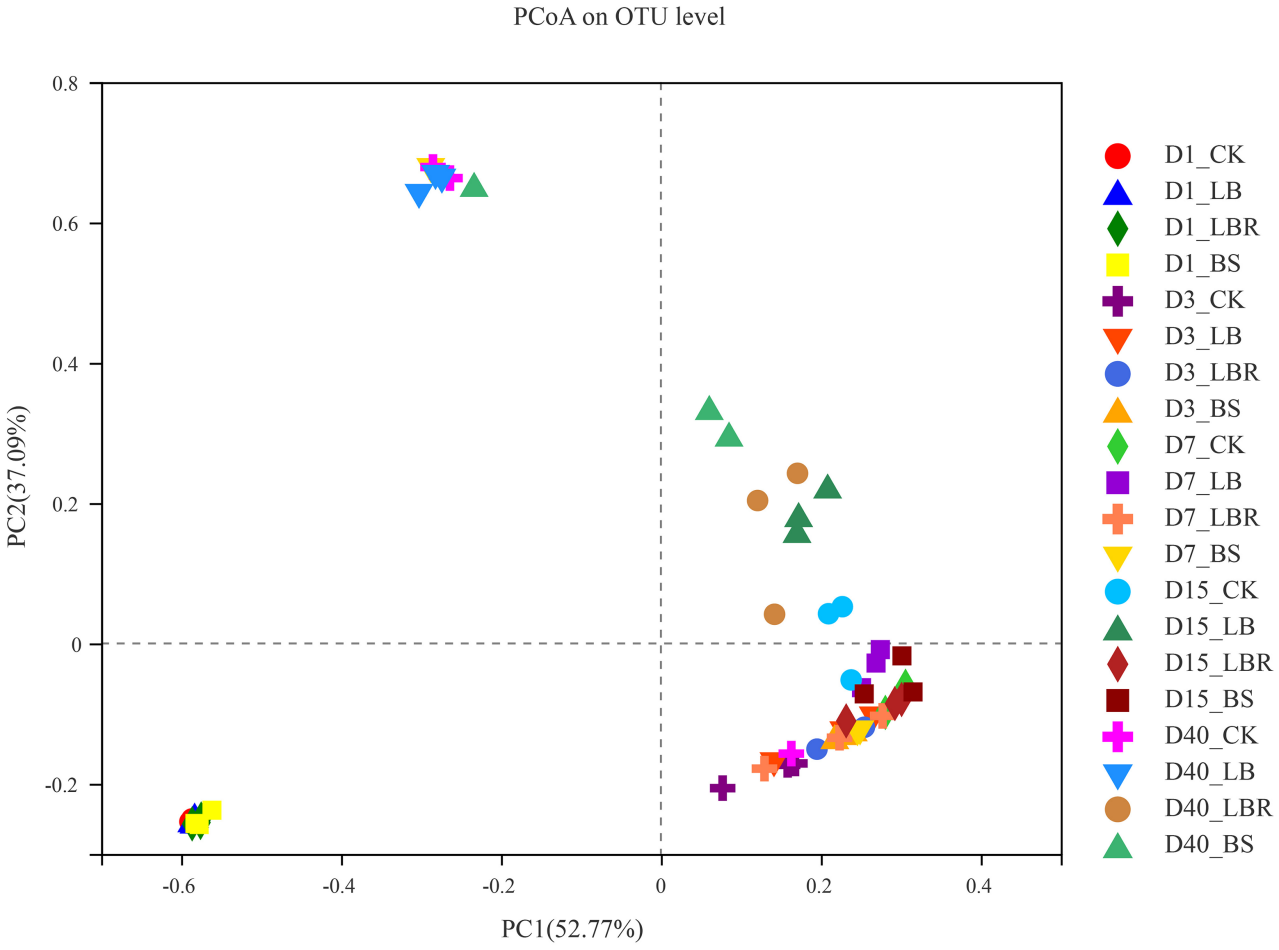
Principal component analysis (PCoA) of bacterial communities in sorghum-sudangrass silage after ensiling for 1, 3, 7, 15, and 40 days (CK, control; LB, *Lactobacillus buchneri*; LBR, *Lactobacillus brevis;* BS, *Bacillus subtilis*).

The bacterial alpha diversity of sorghum-sudangrass silage is shown in Table 4. The Coverage values of all treatment groups were greater than 0.99. Days of ensiling significantly affected the Shannon, Simpson, Ace, and Chao indices (*p* < 0.05), and additives had significant effects on the Ace and Chao values of sorghum-sudan grass silage (*p* < 0.05). The addition of LB had no significant effect on α-diversity during the ensiling process. The Shannon index of the LBR and BS groups was higher than that of the CK group on the first day of fermentation (*p* < 0.05). No significant differences in the Shannon, Simpson, Ace, and Chao indices of silages with additives and the CK were noted after 3 days of ensiling. As shown in Figure 1, principal component 1 (PCoA 1) and component 2 (PCoA 2) could explain 52.77 and 37.09% of the total variance, respectively. A clear separation of bacterial communities of sorghum-sudangrass silage between day 1 of ensiling and day 40 of ensiling could be observed. At 40 days of ensiling, the LBR and BS groups were significantly separated from CK.

**Table 4:** Alpha diversity of bacterial diversity of sorghum-sudangrass silage.

Item	Ensiling days	Treatment				SEM	Significance		
		CK	LB	LBR	BS		*T*	*D*	*T* × *D*
Shannon	1	1.23Ba	1.19Ba	1.36Aa	1.41Aa	0.032	0.050	<0.001	0.436
	3	0.95ab	0.85b	0.84ab	0.85b				
	7	0.45b	0.60b	0.91ab	0.54b				
	15	0.55b	0.31c	1.01ab	0.59b				
	40	0.62b	0.58b	0.65b	0.53b				
Simpson	1	0.45c	0.48c	0.40b	0.36b	0.013	0.092	<0.001	0.271
	3	0.55bc	0.67b	0.68a	0.68a				
	7	0.84a	0.79ab	0.65a	0.80a				
	15	0.80a	0.90a	0.66a	0.81a				
	40	0.75ab	0.79ab	0.77a	0.82a				
Ace	1	91.54ab	70.27	88.43b	110.05ab	4.899	0.008	<0.001	0.177
	3	96.19ab	85.80	89.90b	75.80b				
	7	59.23b	80.38	90.58b	144.65ab				
	15	164.91a	101.65	180.38a	174.79ab				
	40	154.64ABa	107.33B	95.70Bb	192.08Aa				
Chao	1	79.60BCb	63.70C	82.99Bb	106.37A	4.460	0.005	<0.001	0.291
	3	74.16b	75.65	80.38b	65.37				
	7	51.90b	72.53	73.83b	130.74				
	15	113.13ABa	89.54B	175.37Aa	172.52AB				
	40	119.10ABa	92.22B	97.31Bb	145.28A				
Coverage	1	0.9995ABa	0.9996Aa	0.9995ABa	0.9993Bab	0.000	0.036	<0.001	0.454
	3	0.9994a	0.9995a	0.9994a	0.9996a				
	7	0.9996a	0.9995a	0.9995a	0.9992ab				
	15	0.9991b	0.9992b	0.9989b	0.9987b				
	40	0.9990ABb	0.9992ABb	0.9993Aa	0.9989Bab				

*CK, control; LB, Lactobacillus buchneri; LBR, Lactobacillus brevis; BS, Bacillus subtilis. Different capital letters indicate differences between treatment groups at the same time. Different lowercase letters indicate different silage times for the same treatment at the 0.05 level; no letter mean no significant difference. SEM, Standard error of the mean; T, treatment; D, Ensiling days; T × D, The interaction between treatment and ensiling days.*

**Figure 2:**
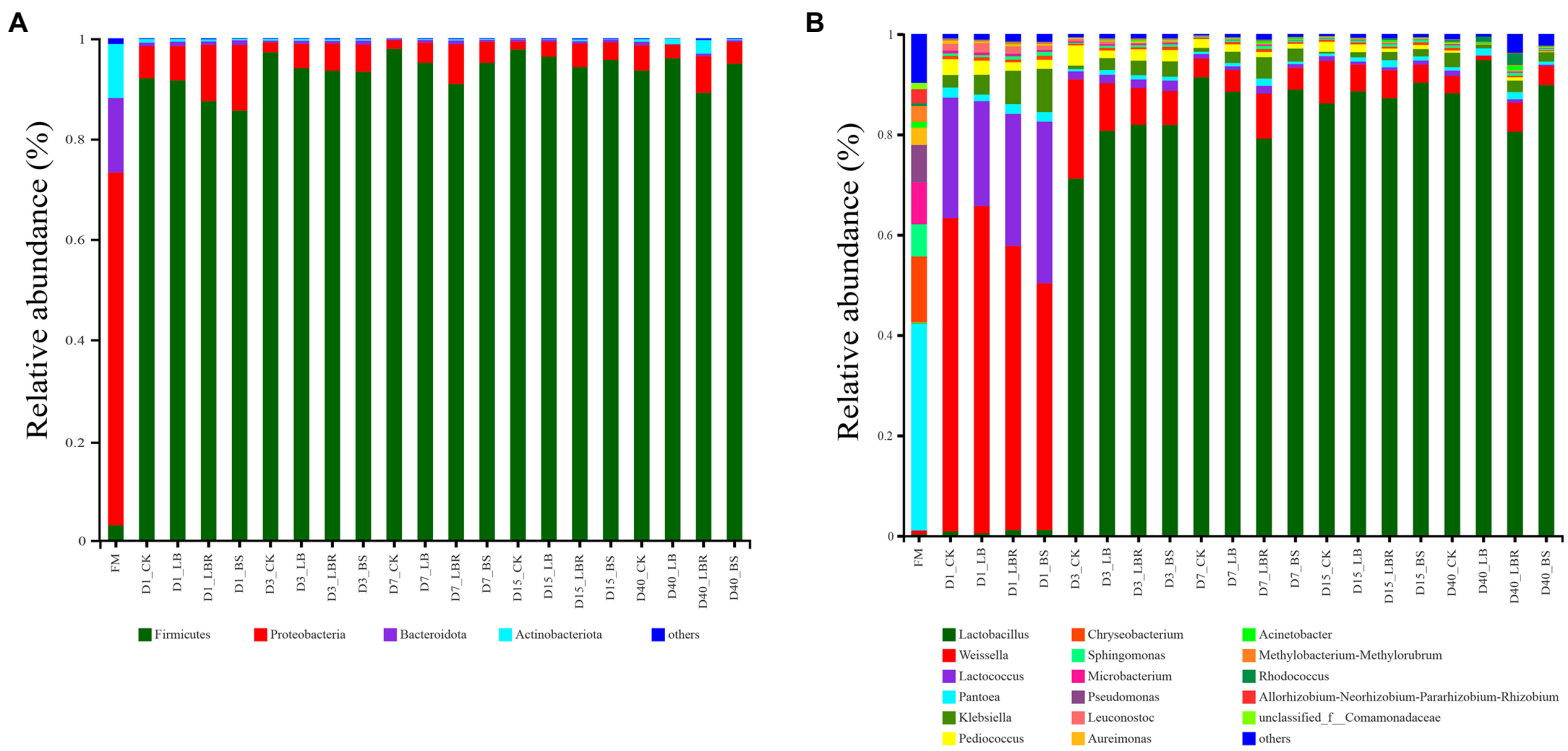
Relative abundance of bacterial communities at the phylum **(A)** and genus levels **(B)** in sorghum-sudangrass silage after ensiling for 1, 3, 7, 15, and 40 days (FM, fresh matter; CK, control; LB, *Lactobacillus buchneri*; LBR, *Lactobacillus brevis;* BS, *Bacillus subtilis*).

At the phylum level, the bacterial community in fresh sorghum-sudangrass was dominated by *Proteobacteria, Firmicutes, Bacteroidota,* and *Actinobacteriota* (Figure 2A). The dominant phylum in the fresh sorghum-sudangrass was *proteobacteria* (70.31%). After fermentation, the dominant bacteria changed to *Firmicutes* (92.02%). At the genus level, the dominant genus in fresh sorghum-sudangrass was *Pantoea* and the relative abundance of *Pantoea* decreased sharply after fermentation (< 5%; Figure 2B). The abundance of *Weissella* in all silages increased rapidly (> 60%) on the first day of ensiling, and the abundance of *Lactobacillus* in all silages increased rapidly (> 70%) on the third day of ensiling and remained high after 3 days of ensiling. *Acinetobacter* abundance was increased in the LB group compared with CK during 3–7 days of ensiling, and *Serratia* abundance was significantly increased in the LB group compared with CK during 7–15 days of ensiling (*p* < 0.05; Supplementary Figure S1). The LBR group exhibited increased *Klebsiella* abundance compared with CK on the first day of ensiling, significantly increased *Acinetobacter* abundance compared with CK during 3–7 days of ensiling, and significantly increased *Serratia* abundance compared with CK during 7–15 of ensiling (*p* < 0.05; Supplementary Figure S1). The BS group exhibited increased *klebsiella* than CK on the first day of ensiling (*p* < 0.05), significantly increased *Acinetobater* compared with CK during 3–7 days of ensiling (*p* < 0.05), and increased *Serratia* abundance compared with CK at 15 days of ensiling. *Aquabacterium*, *Pelomonas*, *Brevundimonas*, and *Ralstonia* levels were significantly increased in both the LBR and BS groups compared with CK at 40 days of ensiling (*p* < 0.05; Supplementary Figure S1).

**Figure 3:**
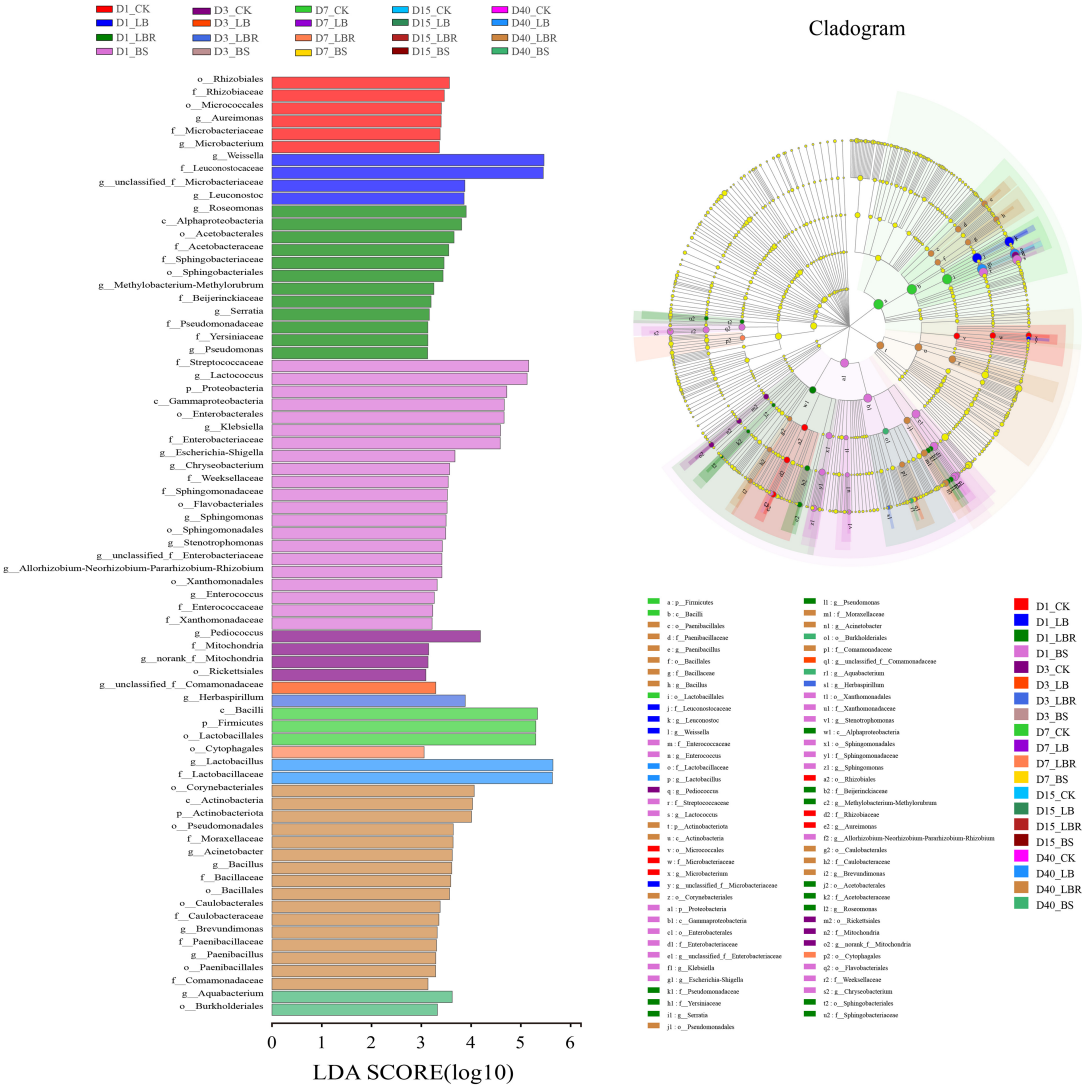
Comparison of microbial variations in sorghum-sudangrass after ensiling for 1, 3, 7, 15, and 40 days using the LEfSe online tool (CK, control; LB, *Lactobacillus buchneri*; LBR, *Lactobacillus brevis;* BS, *Bacillus subtilis*).

The linear discriminant analysis effect size (LefSe) was applied to explore the relative richness (*p* < 0.05, LDA > 3.0) of silages (Figure 3). On the first day of ensiling, *Weissella* and *Leuconostoc* represent differential bacteria in the LB group. In the LBR group, the differential bacteria included *Roseomonas*, *Serratia,* and *Pseudomonas*. In the BS group, the differential bacteria were included *Lactococcus*, *Klebsiella, Eschcrichia-Shigella, Chryseobacterium, Sphingomonas, Stenotrophomonas,* and *Enterococcus.* At 40 days of ensiling, the differential bacteria in the LB group was *Lactobacillus*; the differential bacteria in the LBR group were *Acinetobacter*, *Bacillus*, *Brevundimonas*, and *Paenibacillus*; and the differential bacteria in BS was *Aquabacterium*.

**Figure 4:**
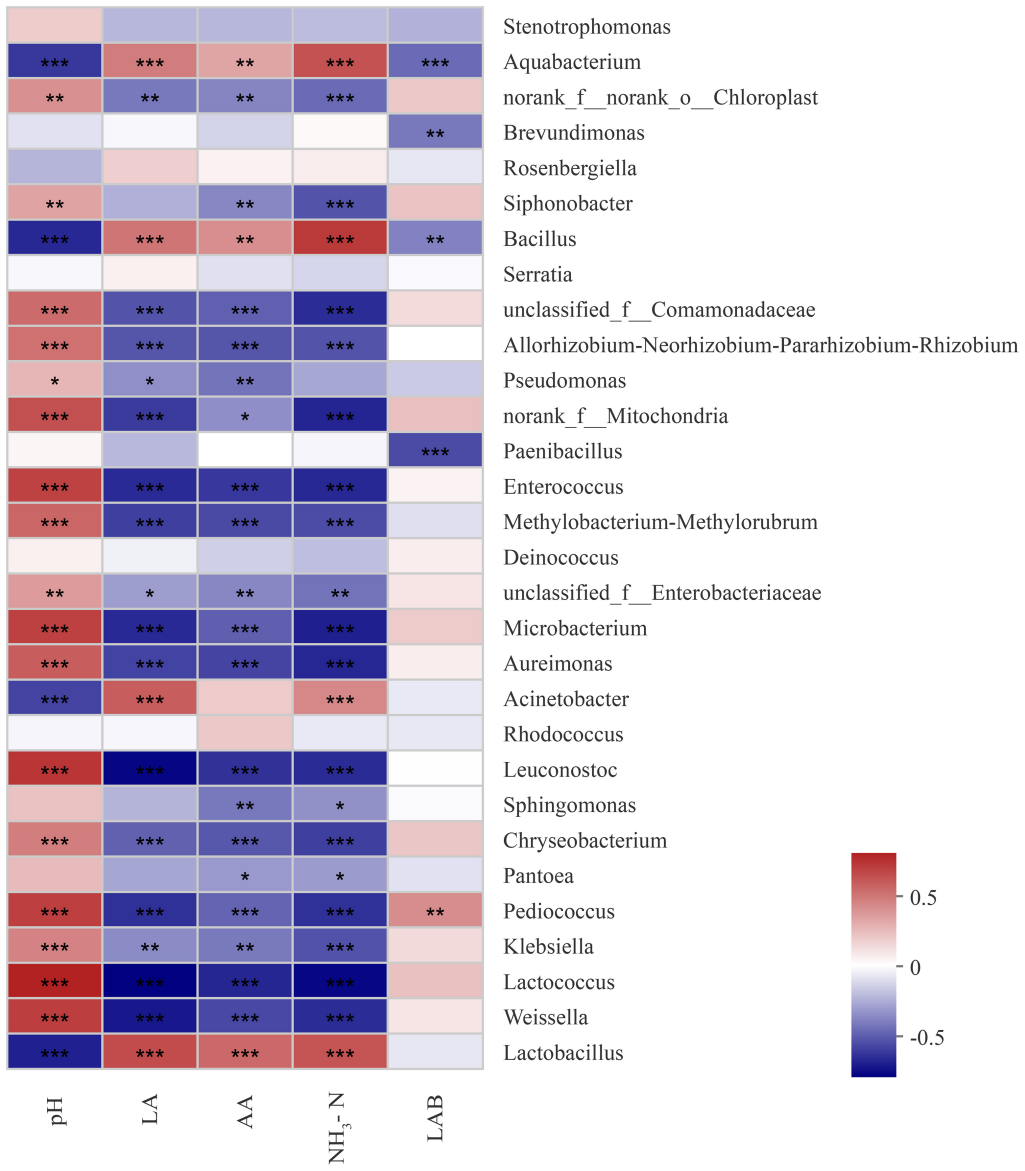
Correlation of the bacterial community and silage fermentation (LA, Lactic acid; AA, Acetic acid; NH_3_-N, ammonia nitrogen; LAB, Lactic acid bacteria; *p*-values are shown as *0.01 < *P* ≤ 0.05, **0.001 < *p* ≤ 0.01, ***p ≤ 0.001).

The relevance between fermentation quality and bacterial communities was assayed and is presented in Figure 4. The pH was positively associated with *Weissella*, *Lactococcus*, *Kliebslella*, *Pediococcus*, *Chryseobacterium*, *Leuconostoc Aurelmonas,* and *Microbacterium* (*p* < 0.05), and negatively related to *Lactobacillus* and *Acinetobacter* (*p* < 0.05). The LA content was positively correlated with *Lactobacillus* and *Acinetobacter,* and negatively correlated with *Weissella Lactococcus*, *Kliebslella, Pediococcus, Chryseobacterium, Leuconostoc, Aurelmonas,* and *Microbacterium*. The AA content was positively correlated with *Lactobacillus* (*p* < 0.05).

### Nitrate, N_2_O and microbial function prediction of silage

**Figure 5:**
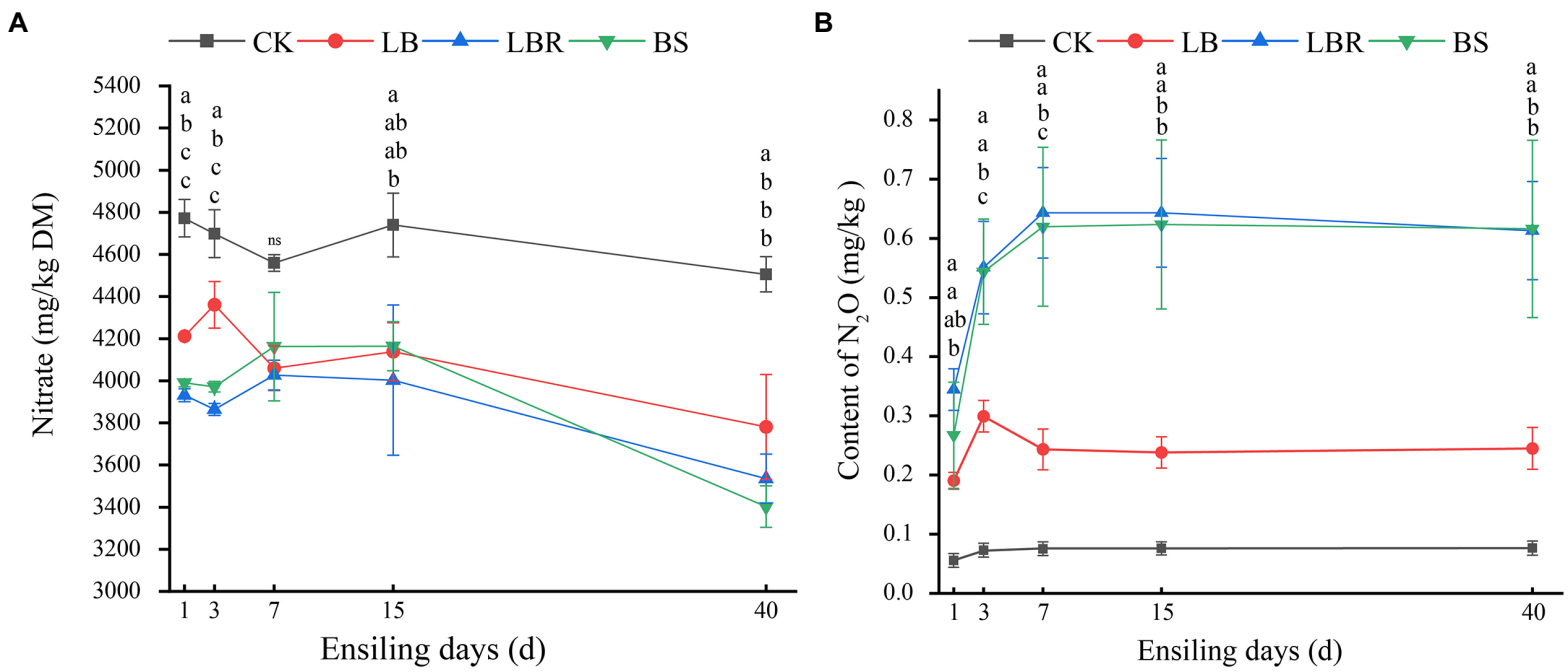
The nitrate content **(A)** and N_2_O **(B)** content in sorghum-sudangrass after ensiling for 1, 3, 7, 15, and 40 days (CK, control; LB, *Lactobacillus buchneri*; LBR, *Lactobacillus brevis;* BS, *Bacillus subtilis;* different lowercase indicate differences between treatment groups at the same time at the 0.05 level; ns, mean no significant difference).

**Figure 6:**
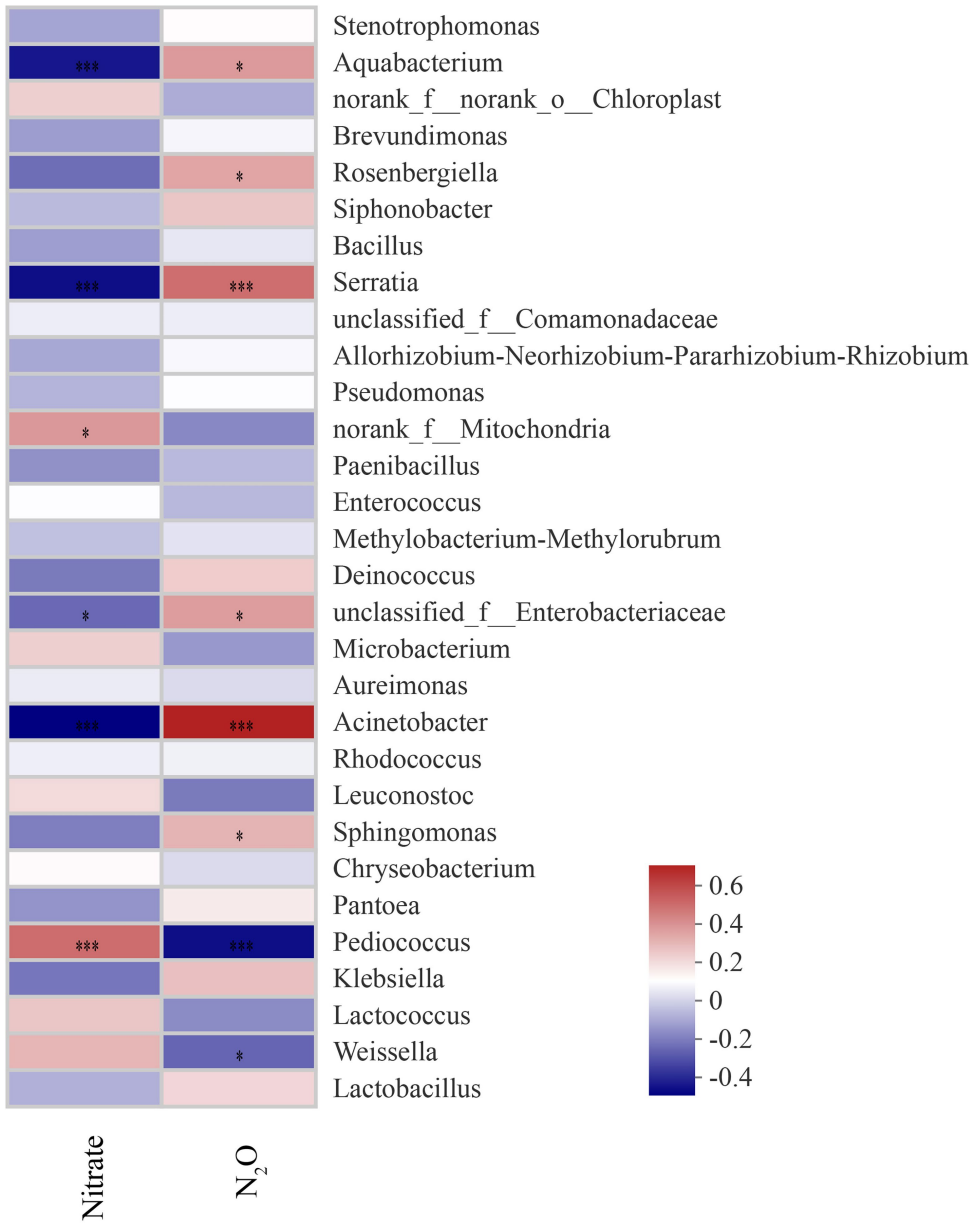
Correlation of bacterial communities with nitrate and N_2_O content (*p*-values are shown as *0.01 < *p* ≤ 0.05, ***p ≤ 0.001).

The content of nitrate in sorghum-sudangrass was significantly influenced by additives and ensiling days (*p* < 0.05; Figure 5A). At 40 days, the nitrate content in the silages with additives was significantly lower than that in the CK group (*p* < 0.05). Additives and days of ensiling had a significant effect on the N_2_O content in sorghum-sudangrass silage (*p* < 0.05; Figures 5B). The LBR and BS groups had significantly higher N_2_O content than CK during ensiling (*p* < 0.05). The LB group had a significantly higher N_2_O content than CK from day 3 to day 7 of ensiling; however, the N_2_O content was not significantly different from CK after 15 days of ensiling (*p* < 0.05). The Nitrate content was positively correlated with *Pediococcus* and *norank*_f_*mltochondria* (*p* < 0.05), and negatively correlated with *Acinetobacter* and *unclassified*_*f*_*enterobacteriaceae* (*p* < 0.05; Figure 6). The N_2_O content was positively correlated with *Acinetobacter* and *unclassified_f_enterobacteriaceae* (*p* < 0.05), and negatively correlated with *Pediococcus* and *norank_f_mltochondria* (*p* < 0.05; Figure 6)*.*

**Figure 7:**
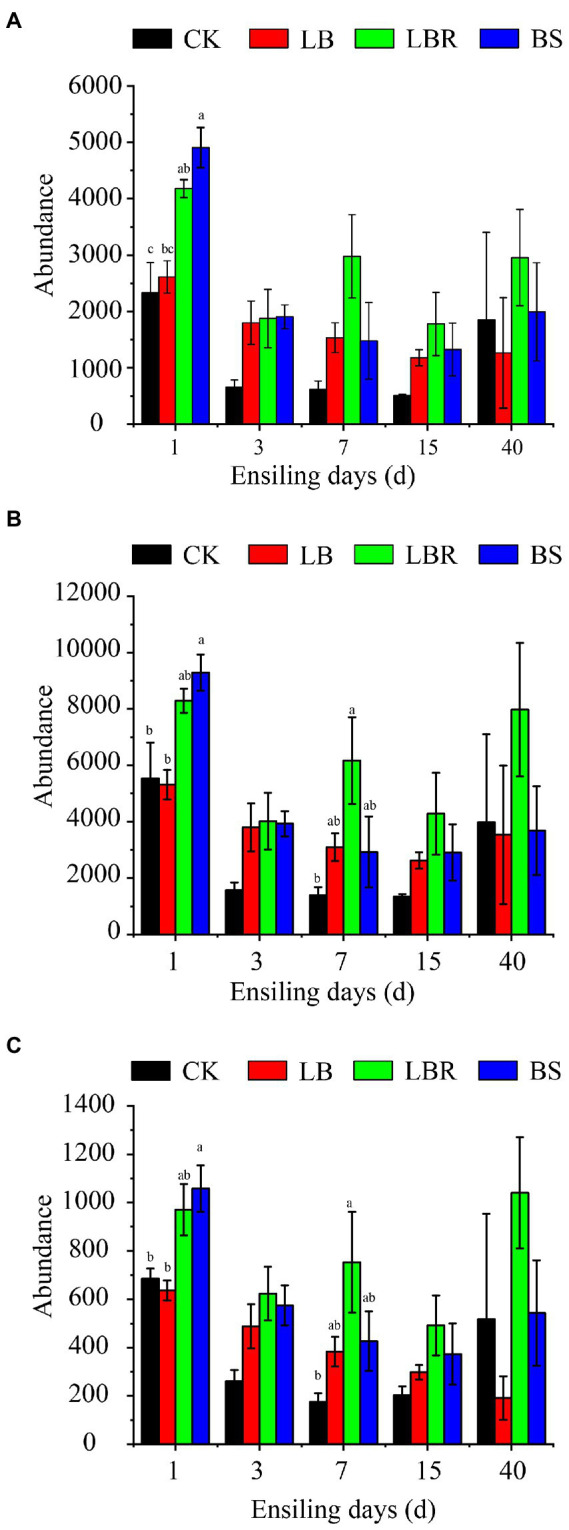
The denitrification **(A)**, dissimilatory nitrate reduction **(B)**, and assimilatory nitrate reduction **(C)** functional profiles of the microbial communities in sorghum-sudangrass after ensiling for 1, 3, 7, 15, and 40 days (CK, control; LB, *Lactobacillus buchneri*; LBR, *Lactobacillus brevis;* BS; *Bacillus subtilis;* different lowercase indicate differences between treatment groups at the same time at the 0.05 level).

The Figure 7 predicts the effect of additives on the nitrate degradation function of bacteria during ensiling. Compared to CK, the metabolic abundance of denitrification increased significantly on the first day of ensiling in the LBR group, and the metabolic abundance of dissimilatory nitrate reduction and assimilatory nitrate reduction increased significantly on day 7 of ensiling (*p* < 0.05). Compared to CK, the BS group showed a higher metabolic abundance of silage denitrification, dissimilatory nitrate reduction, and assimilatory nitrate reduction on the first day of ensiling (*p* < 0.05).

## Discussion

### Characteristics of fresh sorghum-sudangrass

The WSC and epiphytic microorganisms in silage are key factors in determining the silage quality of the forage (Hu et al., 2009). The DM, WSC, and microbial counts of sorghum-sudangrass fulfill the theoretical requirements for well-fermented silage (Li et al., 2019). However, the nitrate content in the fresh sorghum-sudangrass in this experiment was as high as 5520.55 mg/kg DM, which can threaten the health of ruminants and pose a challenge to the safety of sorghum-sudangrass silage (Schrenk et al., 2020).

### Fermentation quality and nutrient composition of silage

The pH of the sorghum-sudangrass silage decreased rapidly, and the LA content increased significantly during the initial 7 days of ensiling, indicating that extensive lactic acid fermentation occurred during the initial 7 days of ensiling. This phenomenon likely occurs because the forages are chopped into short parts to ensure the rapid release of plant juice, thus stimulating the growth of homofermentative lactic acid bacteria in the early period (Liu et al., 2019; Wang S. R. et al., 2022). Compared to CK, the LB group had a lower pH at 3 days of ensiling, whereas the pH was higher at 40 days of ensiling. The pH changes during ensiling were attributed to the accumulation of organic acids (Kung Jr. et al., 2018). In this study, the higher pH of the LB group at the end of ensiling was caused by the addition of LB to promote the accumulation of AA at the later stages of silage (Blajman et al., 2018). The pH was lower in the LBR group after 3 days of ensiling than in the CK. The LA content of the LBR group was significantly higher than that of CK on the third day of ensiling and during 15–40 days of ensiling. Therefore, the addition of LBR is more beneficial to obtain lower pH and higher LA content for sorghum-sudangrass silage. Similarly, lower pH and higher LA content were observed in silage with LBR in the study by Fijalkowska et al. (2020). Bai et al. (2020) reported that antimicrobial peptides produced by *Bacillus subtilis* could promote the fermentation of homozygous lactic acid bacteria. The BS group in our study had a higher LA concentration and lower pH at 40 days of fermentation. Butyric acid and NH_3_-N are both undesirable products in preserving forage (Kung Jr. et al., 2018; Li et al., 2018). In the present study, butyric acid was not detected throughout the sorghum-sudangrass silage, probably because the rapid decrease in pH during fermentation prevented *Clostridium* from fermenting soluble carbohydrates to butyric acid or converting lactic acid to butyric acid (Kung Jr. et al., 2018). The low NH_3_-N content in all groups in this study was due to the low number of undesirable microorganisms during the ensiling of sorghum-sudangrass (Wang et al., 2017). No butyric acid and lower content of NH_3_-N were detected which is beneficial for the quality of sorghum-sudangrass silage. Lactic acid bacteria can ferment a variety of substrates and rapidly produce large amounts of lactic acid for pH decline and microorganism inhibition. (Pang et al., 2012; Yang et al., 2021). The decrease in lactic acid bacteria numbers after 7 days of ensiling was related to insufficient silage substrate and the acidic fermentation environment of silage. At 40 days of ensiling, the higher pH in the LB group had a weaker inhibitory effect on lactic acid bacteria compared to the other group, so the LB group had a higher number of lactic acid bacteria. The number of yeast decreased rapidly after fermentation, which was due to the inhibition of their growth by AA produced after fermentation (Muck et al., 2018).

The nutrient composition of the sorghum-sudangrass silage after the addition of LB and LBR was similar to that of the CK, indicating that the addition of LB and LBR did not significantly alter the nutrient composition of sorghum-sudangrass. Similar findings were observed in the experiments performed by Alhaag et al. (2019) and Wang et al. (2020). Research has shown that the addition of BS inhibits the proteolysis in alfalfa silage and whole plant corn silage (Bai et al., 2020; Bai J. et al., 2022). In this study, the CP content of the BS group was significantly greater than that of the CK at 40 days of ensiling, indicating that the addition of BS to sorghum-sudangrass silage inhibited proteolysis.

### Bacterial community in silage

The Coverage of all samples was 0.99, indicating that the depth of sequencing was sufficient to describe the dynamics of the bacterial community (He et al., 2020). The Shannon index was significantly lower in all treatment groups after 40 days of silage compared to the first day of silage and the Simpson index was significantly higher. As fermentation proceeded, the pH of the silage environment decreased, and the oxygen content gradually decreased to an anaerobic environment. This environment lead to a decrease in anaerobic microorganisms and acid-intolerant epiphytic microorganisms (Yan et al., 2019), resulting in a decrease in the diversity of the bacterial community of sorghum-sudangrass silage. In this study, the addition of LB did not affect α-diversity during the overall fermentation process. The Shannon index with LBR addition and the Shannon and Chao indices with BS addition were higher than those of the CK group on the first day of ensiling. The acidic environment had an inhibitory effect on the harmful microorganisms in silage fermentation (Ellis et al., 2016). On the first day of ensiling, the pH was slightly higher in the LBR and BS groups compared with CK, and both coliform bacteria and yeast counts were higher than in CK, which probably caused the differences in bacterial diversity between the LBR and BS groups and CK. In this study, there was no significant difference between the α-diversity of silages with additives and the CK during 3–40 days of ensiling. Although the dominant species changed for all silages with additives, the bacterial composition did not become simpler during the ensiling period from 3 to 40 days. (Wang et al., 2019; Xu et al., 2021). The plots of principal component analysis clearly reflected the changes of bacterial community during ensiling of sorghum-sudangrass. Storage time can affect the changes of bacterial communities in silage (Guo et al., 2021). The bacterial communities were significantly separated on day 1, days 3–15, and days 40, indicating that ensiling time affected the bacterial community of sorghum-sudangrass silage. Bacterial community changes during silage after the use of additives can indicate differences in the fermentation quality of silage (Ni et al., 2017a). In this study, the LBR and BS groups were significantly separated from the CK at 40 days of ensiling. We hypothesized that the addition of LBR and BS affected the quality of silage by changing the bacterial community structure at the end of ensiling.

In this study, *Proteobacteria* was the most abundant phylum in fresh sorghum-sudangrass, which is consistent with previous studies (Eikmeyer et al., 2013; Wang S. et al., 2022). At the beginning of ensiling (1 day), the bacterial community of all silages was dominated by the *Firmicutes* phylum because the anaerobic conditions and acidic environment formed during ensiling favoured the growth of the *Firmicutes* phylum (Keshri et al., 2018; Ali et al., 2020). *Weissella* is an important heterotypic lactic acid bacterium that initiates early fermentation and that the decrease in pH during silage inhibits its growth (Muck et al., 2018), after ensiling is performed by highly acid-tolerant homotypic fermenting lactic acid bacteria. In our study, *Weissalla* and *Lactococcus* were dominant in all silages on the first day of ensiling, and *Lactobacillus* was dominant in all silages after 3 days of ensiling. In addition, all silages had lower pH and higher lactic acid content, thus explaining the good fermentation quality of the silages (Guan et al., 2018). In the present study, the addition of LB increased the abundance of *Serratia* at 7–15 days of ensiling, and LBR significantly increased the abundance of *Klebsiella* at 1 day of ensiling and *Serratia* at 7–15 days of ensiling. Similarly, the addition of BS significantly increased the abundance of *Klebsiella* at 1 day of ensiling. *Klebsiella* and *Serratia* are usually considered as negative microorganisms for silage fermentation (Sun et al., 2021). However, in the present study, the LB, LBR, and BS were all dominated by *Lactobacillus* after 3 days of ensiling. In addition, the bacteria with the greatest contributions in the LB group included *Weissella* and *Leuconostocaceae*, and the bacteria with the greatest contributions in the BS group were *Lactococcus* and *Streptococcaceae* at 1 day of ensiling. Therefore, the addition of LBR and BS will not have a negative effect on the quality of sorghum-sudangrass silage.

*Lactobacillus* and *Acinetbacter* were significantly negatively correlated with the pH of the sorghum-sudangrass silage, while *Lactococcus*, *Pediococcus*, *Leuconostoc,* and *Enterococcus* were significantly positively correlated. The opposite trend was noted for fermentation acids, including LA and AA. *Lactococcus* starts lactic acid fermentation at the beginning of ensiling, but these species are sensitive to low pH and cannot survive in acidic environments (Ni et al., 2017b; Liu et al., 2019; Yang et al., 2019). Therefore, *Lactobacillus* plays a crucial role in lowering pH in the late silage stage.

### Nitrate, N_2_O and microbial function prediction of silage

At the end of ensiling, the nitrate content in the additive group was significantly lower than that in CK, indicating that the addition of LB, LBR, and BS was beneficial for the degradation of nitrate in sorghum-sudangrass silage. Previous studies have shown that microorganisms, such as *Enterobacteriaceae* and *Lactobacillus,* in silage forage can degrade nitrate (Spoelstra, 1985). Bai C. et al. (2022) demonstrated that *Panobacter* spp., *Pseudomonas* spp., and *Enterobacter* spp. were negatively correlated with the nitrate content of silage sorghum-sudangrass. In addition, the *Acinetobacter*, *Serratia,* and *Aquabacterium* are also able to degrade nitrate in studies based on paddy soil and water process engineering (Zhang et al., 2016; Chen et al., 2021; Shelly et al., 2021). In the present study, nitrate content was negatively correlated with *Acinetobacter*, *Serratia*, *Aquabacterium,* and *unclassified_f_enterobacteriaceae*, indicating that they promoted the degradation of nitrate. In the LB group, *Acinetobacter* and *Serratia* were more abundant than in the CK group at 3–15 days of ensiling. The abundance of *Klebsiella, Acinetobacter*, *Serratia,* and *Aquabacterium* was greater in the LBR and BS groups compared with the CK group during ensiling. Moreover, *Klebsiella* was the differential bacteria in the LBR group, and *Klebsiella* and *Aqubacterium* were the differential bacteria in the BS group using LEfSe. This finding indicates that additives promoted nitrate degradation by affecting the bacterial community structure. N_2_O is one of the essential end products in the nitrate degradation process (Hill, 1999; Driehuis et al., 2018). The N_2_O content was significantly higher in the LBR and BS groups compared with CK at the end of silage, indicating that the addition of LBR, and BS contributed to the degradation of nitrate during ensiling of sorghum-sudangrass. This was also evidenced by the correlation between nitrate and N_2_O and the bacterial community of sorghum-sudangrass silage.

The silage process is mediated by microbial metabolic pathways to convert metabolites or degrade substrates (Xiang et al., 2022). We can estimate the impact of bacterial communities on changes in metabolic pathways during silage by predicting bacterial function. Therefore, this study used the KEGG pathway database PICRUSt2 to predict the function of bacterial communities in nitrate degradation in the silage of sorghum-sudangrass. Bacteria can degrade nitrate through denitrification, catabolism nitrate reduction, and assimilation nitrate reduction pathways (Shi et al., 2014). In this study, the addition of LB had a higher metabolic abundance of denitrification, dissimilatory nitrate reduction, and assimilatory nitrate reduction compared with CK, but no significant effects were noted at 3–15 days of ensiling. The addition of LBR significantly increased the metabolic abundance of denitrification on the first day of ensiling, and significantly increased the metabolic abundance of dissimilatory nitrate reduction and assimilatory nitrate reduction at 7 days of ensiling. The addition of BS significantly increased the metabolic abundance of denitrification, dissimilatory nitrate reduction, and assimilatory nitrate reduction on the first day of ensiling. This information explains why the addition of LBR and BS is more beneficial to the degradation of nitrate in sorghum-sudangrass silage.

## Conclusion

The addition of LB increased the pH and acetic acid content, and the addition of LBR and BS increased the lactic acid content and decreased the pH. Sorghum-sudangrass silage has satisfactory fermentation quality. Additives had a significant effect on the bacterial community, increasing the abundance of microorganisms, such as *Acinetobacter, Serratia,* and *Aquabacterium*, but the fermentation process was still dominated by *Lactobacillus*. The addition of LB, LBR, and BS reduced the nitrate content in the silage of sorghum-sudangrass. *Acinetobacter*, *Serratia*, *Aquabacterium,* and *unclassified_f_enterobacteriaceae* were the main bacteria affecting nitrate degradation during sorghum-sudangrass ensiling. The LBR and BS could increase the metabolic abundance of denitrification, dissimilatory nitrate reduction, and assimilatory nitrate reduction. In conclusion, the use of LB, LBR, and BS additives in the silage of sorghum-sudangrass represents an effective strategy to degrade nitrate while guaranteeing its fermentation quality.

## Data availability statement

The datasets presented in this study can be found in online repositories. The names of the repository/repositories and accession number(s) can be found at: https://www.ebi.ac.uk/ena, PRJNA882255.

## Author contributions

MZ, JS, ZY, CB, and YX designed the study and wrote the manuscript. MZ, HZ, GP, and HY performed the experiments. JS, ZY, CB, and YX reviewed and edited the manuscript. MZ, HZ, GP, and HY analyzed the data. ZY, CB, and YX funded and supervised the experiments. All authors contributed to the article and approved the submitted version.

## Funding

This work was funded by the National Natural Science Foundation of China (nos. 31872422, 31772674, and 32160342), the Shenyang Agricultural University Graduate Student Innovation Cultivation Project (2022YCXS35), the Inner Mongolia Science and Technology Plan (2020GG0049), and the China Agriculture Research System of MOF and MARA.

## Conflict of interest

The authors declare that the research was conducted in the absence of any commercial or financial relationships that could be construed as a potential conflict of interest.

## Publisher’s note

All claims expressed in this article are solely those of the authors and do not necessarily represent those of their affiliated organizations, or those of the publisher, the editors and the reviewers. Any product that may be evaluated in this article, or claim that may be made by its manufacturer, is not guaranteed or endorsed by the publisher.

## Supplementary material

The Supplementary material for this article can be found online at: https://www.frontiersin.org/articles/10.3389/fmicb.2022.1052837/full#supplementary-material

SUPPLEMENTARY FIGURE S1 Differences in bacterial community on genus levels in sorghum-sudangrass silage after ensiling for 1 **(A)**, 3 **(B)**, 7 **(C)**, 15 **(D)**, and 40 **(E)** days.
