# Supplementary figures and images for: Effect of exogenous microorganisms on the fermentation quality, nitrate degradation and bacterial community of sorghum-sudangrass silage
Source: Front Microbiol. 2022 Nov 1;13:1052837. doi: 10.3389/fmicb.2022.1052837 (PMC9664940; doi:10.3389/fmicb.2022.1052837)

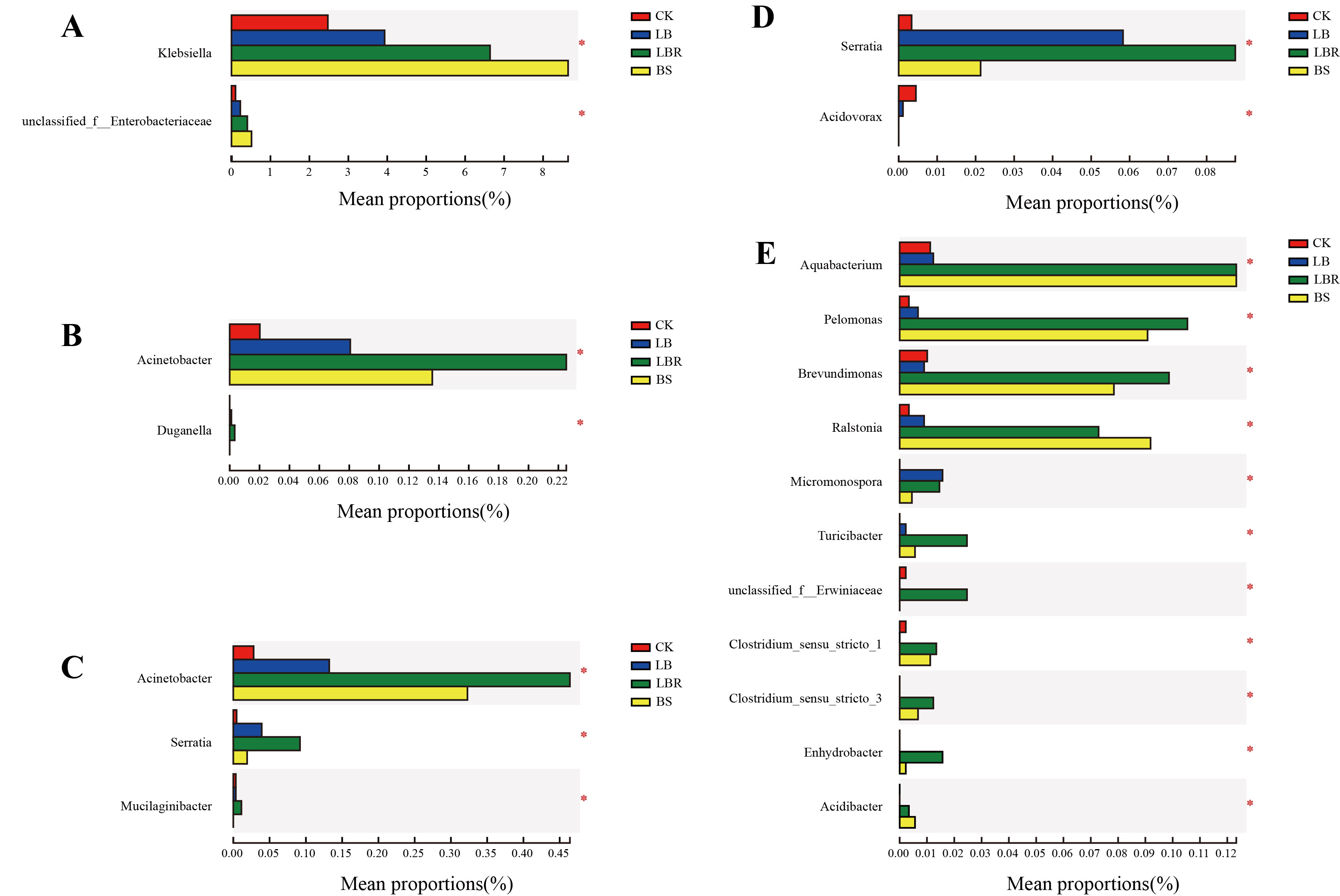

Supplement: SUPPLEMENTARY FIGURE S1 — Differences in bacterial community on genus levels in sorghum-sudangrass silage after ensiling for 1 (A), 3 (B), 7 (C), 15 (D), and 40 (E) days. [file Image_1.JPEG]
